# *Ehrlichia canis* in Human and Tick, Italy, 2023

**DOI:** 10.3201/eid3012.240339

**Published:** 2024-12

**Authors:** Giovanni Sgroi, Nicola D’Alessio, Vincenzo Veneziano, Giuseppe Rofrano, Giovanna Fusco, Mariaelisa Carbonara, Filipe Dantas-Torres, Domenico Otranto, Roberta Iatta

**Affiliations:** Experimental Zooprophylactic Institute of Southern Italy, Portici, Italy (G. Sgroi, N. D’Alessio, G. Rofrano, G. Fusco), University of Naples Federico II, Naples, Italy (V. Veneziano), University of Bari Aldo Moro, Bari, Italy (M. Carbonara, D. Otranto, R. Iatta), Aggeu Magalhães Institute, Recife, Brazil (F. Dantas-Torres); City University of Hong Kong, Hong Kong Special Administrative Region, People’s Republic of China (D. Otranto)

**Keywords:** Ehrlichiosis, *Ehrlichia canis*, rickettsia, ticks, bacteria, tickborne diseases, parasites, zoonoses, Italy

## Abstract

In August 2023, ehrlichiosis was confirmed in a patient in Italy with a *Haemaphysalis punctata* tick attached to his neck. Gene sequences of *Ehrlichia canis* from the tick and the patient were identical, indicating a potential risk for this uncommon infection for persons participating in outdoor activities.

*Ehrlichia canis* (order Rickettsiales, family Anaplasmataceae) is the causative agent of canine monocytic ehrlichiosis and may be incidentally transmitted by brown dog ticks (*Rhipicephalus sanguineus* sensu lato) to a plethora of mammalian hosts, including cats and humans (*1*). In humans, asymptomatic or paucisymptomatic infections have been occasionally reported from the United States (*2*,*3*), Venezuela (*4*,*5*), and Costa Rica (*6*). Despite the risk being relatively uncommon, persons living or visiting environments where ticks and *E. canis* are prevalent in dogs may potentially be at risk for infection (*7*). We report a case of human ehrlichiosis caused by *E. canis* in a patient from Italy, indicating the risk for unconventional tickborne infection in humans participating in outdoor activities in rural areas in Italy.

## The Study

In August 2023, a 42-year-old male patient was referred to the Experimental Zooprophylactic Institute of Southern Italy with a tick attached to his neck. The patient had noticed the tick 48 hours after a hike in a rural area of Salerno Province, Campania region, southern Italy. After obtaining the patient’s signed informed consent, we removed the tick with fine-tipped tweezers, checked the patient’s skin for other ticks, and collected a 7-mL blood sample from his cephalic vein. We used a 2-mL aliquot placed in a Vacutainer (https://www.bd.com) K3-EDTA tube for complete blood count on a CELL-DYN 3700 Hematology Analyzer (Abbott, https://www.abbott.com). We added the remaining 5 mL to a Vacutainer clot activator serum tube for biochemical analysis on a SAT 450 Random Access Analyzer (KPM Analytics, https://www.kpmanalytics.com) after centrifugation for 15 min at 1,500 *g* at room temperature. We classified the tick with regard to developmental stage (larva, nymph, or adult), sex (male or female), and feeding status (engorged or not engorged) by using a Leica MS5 stereomicroscope (https://www.leica-microsystems.com). Then we taxonomically identified the tick by using morphological keys. In addition, we looked for pathogenic microorganisms by performing Romanowsky staining using Diff Stain Quick Kit (ProEko, https://www.proekosrl.com) on smears of the gut, hemolymph, and salivary glands of the tick and peripheral blood of the patient.

We extracted DNA from the tick and the patient’s blood by using QIAamp DNA Blood and Tissue kit (QIAGEN, https://www.qiagen.com) and molecularly tested it for tickborne pathogens (*8*,*9*) (Table 1). The tick was also molecularly identified at species level by the amplification of a 248-bp partial fragment of the 16S rRNA gene, with forward (5′-CTGCTCAATGATTTTTTAAATTGCTGT-3′) and reverse (5′-TTACGCTGTTATCCCTAGAG-3′) primers, by using the following thermocycling conditions: 95°C for 10 minutes of initial denaturation followed by 35 cycles of 94°C for 45 seconds, 58°C for 60 seconds, 72°C for 60 seconds, and 72°C for 7 minutes of final extension. We ran all PCRs in a final volume of 50 μL including 5 μL of 10× PCR buffer II, 6 μL of 25 mmol MgCl_2_, 5 μL of 1.25 mmol of dNTPs, 0.5 μL of 100 pmol/μL of each primer, and 1.25 U of AmpliTaq Gold (Applied Biosystems, https://www.thermofisher.com). We sequenced the purified amplicons in both directions by using a BigDye Terminator v3.1 Cycle Sequencing Kit in a 3130 Genetic Analyzer (Applied Biosystems), then used Geneious version 9.0 (https://www.geneious.com) for editing and analysis. We compared the resulting sequences with those available in the GenBank database by using Nucleotide BLAST (https://blast.ncbi.nlm.nih.gov/Blast.cgi) and performed the phylogenetic analysis by using the maximum-likelihood method based on the general time reversible model with gamma distribution to assess evolutionary differences among sites (+G) selected by best-fit model (*10*) with MEGA X software (*11*). Our study was approved by the Experimental Zooprophylactic Institute of Southern Italy within the framework of a memorandum of agreement (authorization no. IZSM-DIMBA/23) with the Interdisciplinary Department of Medicine, University of Bari Aldo Moro, according to national regulations.

**Table 1:** Targeted pathogens and related PCR protocols used in study of *Ehrlichia canis* in human and tick, Italy, 2023

Pathogen	Target gene	Primer name	Primer sequence, 5′→3′	Amplicon length, bp	Reference
*Anaplasma*, *Ehrlichia*, *Candidatus* Neoehrlichia spp.	16S rRNA	EHR16-SD EHR16-SR	GGTACCYACAGAAGAAGTCC TAGCACTCATCGTTTACAGC	345	(*8*)
*Ehrlichia canis*	*groEL*	Ehr-groel-F Ehr-groel-R	GTTGAAAARACTGATGGTATGCA ACACGRTCTTTACGYTCYTTAAC	590	(*9*)
*Babesia*, *Theileria* spp.	18S rRNA	RLB-F RLB-R	GAGGTAGTGACAAGAAATAACAATA TCTTCGATCCCCTAACTTTC	460–520	(*8*)
*Borrelia burgdorferi* sensu lato complex	*Flagellin*	FLA1 FLA2	AGAGCAACTTACAGACGAAATTAAT CAAGTCTATTTTGGAAAGCACCTAA	482	(*8*)
*Coxiella burnetii*	IS1111a	Trans-1 Trans-2	TATGTATCCACCGTAGCCAGT CCCAACAACACCTCCTTATTC	687	(*8*)
*Rickettsia* spp.	*glt*A	CS-78F CS-323R	GCAAGTATCGGTGAGGATGTAAT GCTTCCTTAAAATTCAATAAATCAGGAT	401	(*8*)

We identified the tick removed from the patient as an engorged female *Haemaphysalis punctata* (GenBank accession no. PP419005). According to PCR testing, the tick and the patient’s blood scored positive for a fragment of the *E. canis* 16S rRNA gene. The sequences we obtained (GenBank accession nos. OR518413 and OR506261) were identical to each other (100% query cover) and to those of *E. canis* obtained from a red fox (*Vulpes vulpes*) in the same study area, humans from United States and Venezuela, and dogs from Mediterranean Basin countries (Italy, Greece, Israel, Egypt, and Turkey) (Figure). We confirmed the molecular identification of *E. canis* from the tick and the patient’s blood by amplifying the *groEL* gene. The sequences obtained were identical to each other (GenBank accession no. PP839296). Other pathogens were not detected by PCR or cytologic examination of stained smears. No major clinicopathologic abnormalities were detected in the patient, except severely increased alanine aminotransferase and mildly decreased total leukocyte count and aspartate aminotransferase (Table 2). Three days after the first visit, the patient complained of mild symptoms (i.e., fever of 38°C, headache, muscle pain, and malaise) which spontaneously healed within a week, without antimicrobial drug treatment.

**Figure:**
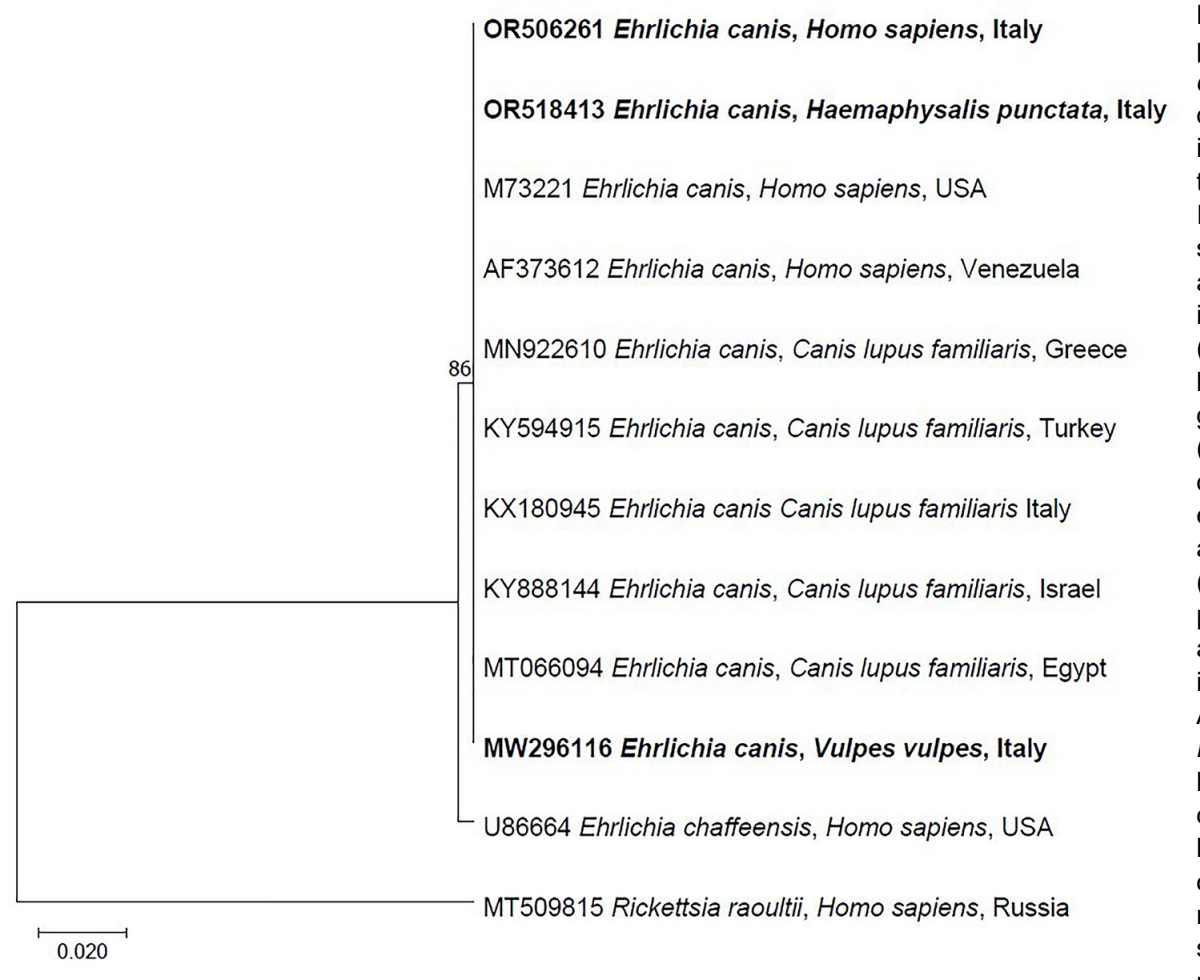
Maximum-likelihood phylogenetic tree of *Ehrlichia canis* 16S rRNA sequences detected in a patient’s blood and in a *Haemaphysalis punctata* tick removed from the patient in Italy, 2023. Boldface indicates sequences amplified in the study area. The tree was inferred including 12 partial sequences (281 bp) under the maximum-likelihood method based on the general time reversible model (*10*) and a discrete gamma distribution was used to model evolutionary rate differences among sites (5 categories) (+*G*, parameter = 0.3727). The percentage of trees in which the associated taxa clustered together is shown next to the branches. A consensus sequence of *Rickettsia raoultii* (MT509815) in a human from Russia was used as outgroup. The tree with the highest log likelihood (−559.69) is shown, obtained from 1,000 bootstrap replications with MEGA X software (*11*). Scale bar indicates nucleotide substitutions per site.

**Table 2:** Complete blood count and serum chemistry for patient positive for *Ehrlichia canis* infection, Italy, 2023*

Parameter	Value (reference range)
Hemoglobin, g/dL	15.0 (13.5–18.0)
Platelets, 10^3^/μL	247.0 (150.0–400.0)
Mean platelet volume, fL	7.8 (6.0–11.5)
Leukocytes, 10^3^/μL	**3.7 (4.0–10.0)**
Erythrocytes, 10^6^ μL	4.9 (4.5–5.9)
Hematocrit, %	46.8 (41.0–53.0)
Mean corpuscular volume, fL	94.6 (80.0–100.0)
Mean corpuscular hemoglobin, pg/dL	30.3 (26.0–34.0)
Mean corpuscular hemoglobin concentration, g/dL	32.0 (31.0–37.0)
Red cell distribution width, %	13.7 (11.5–14.5)
Hemoglobin distribution width, %	2.6 (2.0–3.2)
Neutrophils, %	43.1 (40.0–74.0)
Lymphocytes, %	48.0 (19.0–48.0)
Monocytes, %	5.7 (3.4–9.0)
Eosinophils, %	1.2 (0–8.0)
Basophils, %	1.0 (0–1.5)
Urea, mg/dL	33.0 (20.0–50.0)
Creatinine, mg/dL	1.2 (0.7–1.3)
Cholesterol, mg/dL	198.0 (<200)
High-density lipoprotein-cholesterol, mg/dL	70.0 (40.0–150.0)
Triglycerides, mg/dL	86.0 (<150)
Aspartate aminotransferase, UI/L	**36.0 (<34)**
Alanine aminotransferase, UI/L	**122.0 (10.0–49.0)**

*Boldface indicates out of reference range. fL,femtoliter.

## Conclusions

Data suggest that *E. canis* may infect persons bitten by *H. punctata* ticks in Europe, which may represent a potential risk for persons participating in outdoor activities (e.g., hiking), where those ticks are commonly found on vegetation from spring to autumn in southern Italy (*12*). The finding of *E. canis* DNA in the tick removed from the *E. canis*–positive patient indicates the potential involvement of *H. punctata* ticks in transmission of the pathogen. Although no experimental evidence of the competence of this tick species for transmitting *E. canis* is available, circumstantial evidence suggests its participation as a vector. For instance, in southern Italy, *H. punctata* ticks parasitize humans (*7*) and harbor *E. canis* DNA (*13*).

The similarity of *E. canis* sequences from the tick and patient with those of foxes from the same study area (GenBank accession no. MW296116) suggests circulation of the same *E. canis* genotype among dogs, humans, and wildlife. A previous analysis of the *E. canis* TRP36 gene sequences from different countries revealed the occurrence of the US genogroup in foxes from southern Italy (*14*). The US genogroup is the most common genotype found in dogs and ticks in Eurasia (*14*). The role of foxes as wild reservoirs of *E. canis* needs to be interpreted with caution, especially considering that dogs are the principal reservoirs of this bacterium. The absence of *E. canis* inclusions in the stained smears from the tick and the patient was somewhat expected considering the low sensitivity of the method, even for detecting morulae of other ehrlichial species that are more frequently found in human patients (*15*). The patient’s clinicopathological abnormalities (i.e., decreased leukocytes and aspartate transaminase and increased alanine transaminase) and clinical signs and symptoms (i.e., fever of 38°C, headache, muscle pain, and malaise) are in accordance with previous data (*2*,*5*). Future molecular surveys assessing the circulation of *E. canis* in persons, dogs, and wildlife exposed to ticks should ultimately increase awareness about this zoonosis and be used to establish proper strategies to mitigate the risk for transmission.
